# 

**Published:** 1903-06

**Authors:** 


### CONTENTS.

ORIGINAL COMMUNICATIONS—

UNCINARIASIS (ANKYLOSTOMIASIS); ITS FREQUENCY AND IMPORTANCE IN THE SOUTHERN STATES. By H. F. Harris, M.D., Atlanta, Ga. .... 133

POST-OPERATIVE TREATMENT OF SIX CASES OF ABSCESS OF THE APPENDIX, WITH SOME OBSERVATIONS ON THE USE OF LARGE DOSES OF STRYCHNIN FOR ILEUS. By R. M. Harbin, M.D., Rome, Ga. .... 148

THE TREATMENT OF STRICTURE OF THE ANTERIOR URETHRA. By W. L. Champion, M.D., Atlanta, Ga. .... 155

MEDICAL JURISPRUDENCE. By James R. Cain, Associate Member Chatham County Medical Society .... 158

EDITORIAL NOTES AND COMMENTS—

THE SUBSTITUTING DRUGGIST .... 165

DR. HENRY MCHATTON .... 168

DR. JAMES H. MCDUFFIE .... 169

NEWS AND NOTES .... 172

CONTENTS CONTINUED .... X

